# Short-Term Cardiovascular Compensatory Responses to Varying Levels of Orthostatic Stress During Active Standing in Older Adults

**DOI:** 10.3390/jcm14207202

**Published:** 2025-10-13

**Authors:** Dihogo Gama de Matos, Jefferson Lima de Santana, Felipe J. Aidar, Stephen M. Cornish, Gordon G. Giesbrecht, Albená Nunes-Silva, Satish R. Raj, Roman Romero-Ortuno, Todd A. Duhamel, Rodrigo Villar

**Affiliations:** 1 Cardiorespiratory & Physiology of Exercise Research Laboratory, Faculty of Kinesiology and Recreation Management, University of Manitoba, Winnipeg, MB R3T 2N2, Canada; dematosd@myumanitoba.ca (D.G.d.M.);; 2 Graduate Program in Physical Education and Kinesiology and Physiological Sciences, Federal University of Sergipe, Sao Cristovao 49100-000, Brazil; fjaidar@gmail.com; 3 Centre on Aging, University of Manitoba, Winnipeg, MB R3T 2N2, Canada; stephen.cornish@umanitoba.ca; 4 Faculty of Kinesiology and Recreation Management, University of Manitoba, Winnipeg, MB R3T 2N2, Canada; 5 Departments of Anesthesia and Emergency Medicine, University of Manitoba, Winnipeg, MB R3T 2N2, Canada; 6 Laboratory of Inflammation and Exercise Immunology, Department of Physical Education, School of Physical Education, Federal University of Ouro Preto, Ouro Preto 35400-000, Brazil; 7 Libin Cardiovascular Institute, Cumming School of Medicine, University of Calgary, Calgary, AB T2N 1N4, Canada; 8 Discipline of Medical Gerontology, School of Medicine, Trinity College Dublin, D02 PN40 Dublin, Ireland; 9 Institute of Cardiovascular Sciences, St. Boniface General Hospital Albrechtsen Research Centre, Winnipeg, MB R2H 2A6, Canada; 10 Department of Physiology and Pathophysiology, University of Manitoba, Winnipeg, MB R3T 2N2, Canada

**Keywords:** systolic blood pressure, diastolic blood pressure, older adults, active standing, orthostatic hypotension

## Abstract

**Background:** The cardiovascular system of older adults is significantly impacted by aging, contributing to blood pressure (BP) dysregulation, particularly during postural transitions. This study compared the short-term cardiovascular compensatory responses of younger adults (YA) and older adults (OA) during sit-to-stand and lie-to-stand. **Methods:** Participants underwent two active standing orthostatic stress tests, involving 5 min of sitting or 10 min of lying, followed by up to 7 min of standing. Beat-to-beat cardiovascular parameters were assessed using a Finometer (Finapres Medical Systems). Systolic (SBP), diastolic (DBP), mean arterial pressure (MAP), cardiac output (CO), stroke volume (SV), systemic vascular resistance (SVR), and HR were measured at baseline, immediately on standing, and throughout four specific phases after standing: phase 1 (0–30 s), phase 2 (30–60 s), phase 3 (60–80 s), and phase 4 (300–420 s). CO-SVR matching was evaluated to assess BP regulation timing. **Results**: Compared to YA, OA exhibited higher SBP, DBP, MAP, and SVR but lower HR, CO, and SV at baseline. Immediately on standing, OA experienced a greater drop in SBP, DBP, MAP, and SVR, blunted HR, reduced CO, and higher SV. The short-term compensatory responses were delayed (30–60 s), particularly in lie-to-stand, due to a transient CO and SVR mismatch observed in phase 1 and subsequent BP stabilization from phases 2–4. **Conclusions**: OA exhibited short-term compensatory cardiovascular dysregulation, particularly during the transition from a lying to a standing position.

## 1. Introduction

The stress induced by performing daily activities, such as getting up from bed or a chair, presents significant physiological challenges due to the abrupt gravitational shift in blood distribution. These challenges demand rapid, coordinated, and integrated dynamic adaptive cardiovascular responses to maintain cerebral blood perfusion and flow [1,2]. On standing, blood pressure (BP) transiently drops, and cerebral blood flow decreases, requiring immediate compensatory adjustments to stabilize hemodynamic responses [3]. Effective dynamic control and regulation of the cardiovascular system is, therefore, critical for maintaining homeostasis and functionality when facing these external challenges [1]. Failure to properly regulate these responses can result in homeostatic dysregulation [4,5,6], commonly observed with advancing age. As we become older, the efficiency of these compensatory responses diminishes, often resulting in impaired BP control and regulation, as well as reduced cerebral perfusion [7]. One common manifestation of this dysregulation is orthostatic hypotension (OH), which is associated with lightheadedness, falls, fractures, and increased risk of cognitive decline [8,9].

While the age-related differences in orthostatic tolerance and the prevalence of orthostatic hypotension are well established [10,11,12], much less is known about the temporal dynamics of cardiovascular compensation during postural transitions. Most research has relied on single-time-point measurements or broad time windows (e.g., 1 min average) but short durations (e.g., 3 min) [13,14,15], overlooking the fine-grained and time-sensitive dynamics of cardiovascular compensation. This is an important limitation since distinct phases of BP regulation unfold immediately after standing and across several minutes following orthostatic stress. Transient instabilities in these phases cannot be detected by conventional assessments and may have clinical significance and consequences [6,16]. For example, delayed or incomplete recovery of BP within the first 15–30 s after standing has recently been linked to increased fall risk and fractures in older adults [17]. While younger adults typically achieve rapid coordination between cardiac output (CO) and systemic vascular resistance (SVR) within the first seconds after standing [3], this CO-SVR matching has not been systematically examined in older adults, who may be particularly vulnerable to phase-specific impairments and at higher risk of OH [12].

Based on these previous gaps, we hypothesized that (1) older adults will show reduced and delayed responses in systolic BP (SBP), diastolic BP (DBP), mean arterial pressure (MAP), SVR, heart rate (HR), and CO responses, particularly during early phases after standing; (2) stroke volume (SV) values will be higher in older adults across all phases, reflecting compensatory reliance on SV due to attenuated HR response; and (3) CO-SVR matching will be delayed in older adults. Therefore, the current study aimed to describe and compare short-term cardiovascular compensatory responses between younger adults (18–30 years) and older adults (60–79 years) during sit-to-stand (lower orthostatic stress) and lie-to-stand (higher orthostatic stress).

## 2. Materials and Methods

### 2.1. Study Design

This is an observational cross-sectional study that used the Strengthening Reporting of Observational Studies in Epidemiology (STROBE) [18] as a reporting guideline.

### 2.2. Settings

This study was conducted at the Applied Research Centre, Faculty of Kinesiology and Recreation Management, University of Manitoba, from August 2022 until August 2023. The study received approval from the University of Manitoba Health Research Ethics Board (approval number—HE2022-0058).

### 2.3. Participants

Participants were recruited using a convenience sampling strategy from Dr. Todd Duhamel’s database (University of Manitoba), which contains ~1000 females who volunteered in the Women’s Advanced Risk-assessment in Manitoba (WARM) Hearts study; the Centre on Aging database (University of Manitoba); visits to Men’s Shed (community hubs where males gather to share skills, learn, and support each other in a welcoming environment); and retirement/care homes located in Winnipeg, Canada. Phone calls, emails, social media platforms (Facebook, Instagram, X), ads, flyers, and posters were also used as recruitment strategies, particularly for younger adults. People interested in participating contacted researchers by email or phone.

Eligibility was determined using a customized screening questionnaire designed by our research laboratory. Participants were included in the study if they were younger (18–30 yrs) or older (60–79 yrs) adults and identified their sex at birth as female or male. Participants were excluded if they had a history of ischemic heart disease, acute myocardial infarction, stroke, percutaneous coronary intervention, coronary artery bypass surgery, congestive heart failure, psychiatric disorders, severe cognitive impairment, or pregnancy. Common age-related conditions, such as hypertension, were not exclusionary and are reported in the results section.

**Figure 1:**
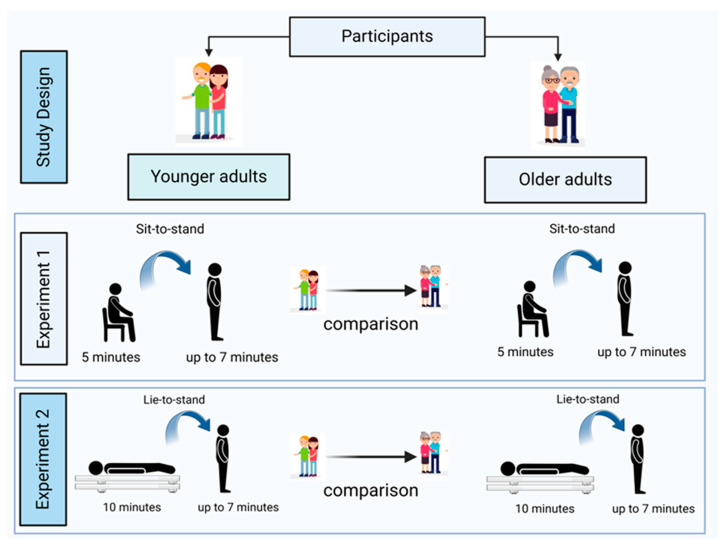
Illustration of the research design and experimental conditions (1 and 2).

All participants completed both the sit-to-stand (Experiment 1) and lie-to-stand (Experiment 2) orthostatic stress challenges in a randomized and counterbalanced order (Figure 1). Variables regarding baseline, immediate responses on standing, dynamic short-term cardiovascular compensatory responses, and CO-SVR matching were compared between younger adults and older adults in Experiments 1 and 2. Participants received pre-instructions 48 h before the lab visit. On the day of this visit, they read and signed an informed consent form after having all their questions and/or concerns addressed by the researchers.

### 2.4. Data Collection

Prior to testing, measurements of resting HR and BP were taken by a trained researcher to ensure participants’ safety, following the procedures adopted by the Canadian Society for Exercise Physiology (CSEP) guidelines [19]. After consent and familiarization, participants underwent active standing orthostatic stress challenges with non-invasive beat-to-beat BP monitoring (Finometer^TM^ Pro Device, Finapres Medical System BV, Amsterdam, The Netherlands). The study was performed in a quiet room with a temperature of 22.0 ± 1.0 °C and humidity and barometric pressure at ambient between 8:30 and 11:30 am on weekdays to minimize circadian cycle influence.

### 2.5. Demographic and Anthropometric Measurements

Demographic (sex assigned at birth, age, and date of birth), anthropometric, and body composition measurements were collected to characterize the sample. Measurements included body mass (kg) and height (m) assessed using an InBody© 270 device (InBody Co., Ltd., Cerritos, CA, USA) and a SECA mobile stadiometer (SECA, Frankfurt, Germany), respectively. Medication used was recorded through participants’ self-reports but was not controlled, and more details were provided in the results section. None of the participants reported being on hormone replacement therapy.

### 2.6. Assessment of Cardiovascular Responses

HR was recorded continuously on a beat-to-beat basis using a 3-lead electrocardiogram (ECG) module (Finapres Medical System, Arnhem, The Netherlands) at a sampling frequency of 1 kHz, with HR derived from R-R intervals. Continuous non-invasive beat-to-beat measurements of SBP, DBP, and MAP were obtained from the arterial pressure signal (photoplethysmography) at the middle finger of the left hand (Finometer, Finapres Medical System, Arnhem, The Netherlands) [20]. The device was calibrated (return-to-flow method), finger circumference was measured to ensure the correct cuff size, and a height correction system was adjusted for hand position relative to heart level. SV was estimated using the Modelflow algorithm (Finometer, Finapres Medical System, Arnhem, The Netherlands) [21]. CO was derived by HR and SV (CO = SV × HR), and systemic vascular resistance (SVR) was derived by dividing MAP by CO (SVR = MAP/CO).

### 2.7. Sample Size

The sample size was determined a priori using the G*Power software (3.1.9.6) (Heinrich Heine University, Dusseldorf, Germany) [22], based on data from an internal pilot study, involving 16 participants (8/group). The power analysis was conducted using a *t*-test for the difference between two independent group means (younger adults vs. older adults) obtained from the amplitude of the overshoot or undershoot measured within 30 s after the nadir. The analysis used an α level of 0.05 and demonstrated an actual power of 0.80 with an effect size of 0.69, derived from the mean ± SD values for younger adults (6.83 ± 6.96 mmHg) and older adults (2.36 ± 5.93 mmHg), resulting in an estimated total sample of 54 participants (27/group). A larger sample of older adults was intentionally collected to account for the increased likelihood of data loss in this population due to signal artifacts and noise, particularly during dynamic postural tasks. Oversampling was used to ensure that adequate statistical power and to retain sufficient data for the planned analyses.

### 2.8. Data Analysis

Data from the Finometer were acquired and processed on a beat-to-beat basis using LabChart 8.0 (ADInstruments, Colorado Springs, CO, USA). ECG data were filtered with a 5–30 Hz bandpass to detect R peaks, except for the 30 s before and 90 s after standing, during which the raw signal was preserved, and Finometer calibration was manually disabled. When ECG traces were of poor quality, analysis was performed on the pressure waveform peaks. BP was calibrated using standard procedures described by the company, followed by a manual calibration (automated BP monitor—ARSIMAI, BSX516, Munster, Germany). Data was saved and transferred to a custom Matlab app for further analyses (Matlab R2024; The MathWorks Inc., Natick, MA, USA). In the Matlab app, noise and motion artifacts were removed using a median filter followed by a low-pass filter (cut-off = 0.05 rad/sample based on scalogram analysis) and a 5 s moving average to smooth the signal and extract parameters of interest [23,24]. Then, the data was interpolated and corrected for delays introduced by resampling at 1 Hz.

### 2.9. Variables Extracted and Analyzed During Active Standing

**Figure 2:**
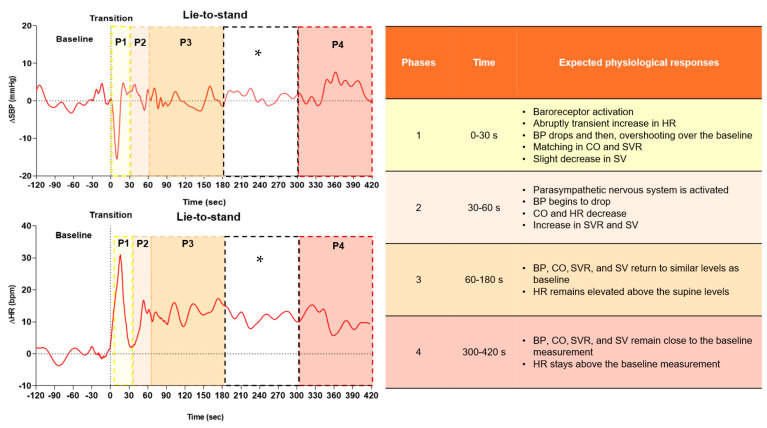
Example of systolic blood pressure (ΔSBP, **top**) and heart rate (ΔHR, **bottom**) responses to lie-to-stand divided into 4 phases. Phase 1: 0–30 s, phase 2: 30–60 s, phase 3: 60–180 s, and phase 4: 300–420 s after transition. * The unshaded interval (180–300 s) represents a stabilization period similar to phase 3; therefore, it was excluded from the analysis. However, phase 4 continues in the analysis as it represents a higher incidence of delayed orthostatic hypotension [26,27].

Baseline values were determined as an average of 2 min before standing. In the first 30 s after standing, the lowest values of SBP, DBP, MAP, and SVR and the highest values of HR, CO, and SV were analyzed. Peak values were defined as the highest value reached within 30 s following standing. Short-term cardiovascular compensatory responses were defined as the highest point reached after the drop. These responses were divided into four specific phases, as follows: phase 1 (0–30 s), phase 2 (30–60 s), phase 3 (60–180 s), and phase 4 (300–420 s), reflecting cardiovascular regulation after active standing orthostatic stress. This phased approach is based on expected physiological responses in healthy adults (reference response) and specific times regarding OH classifications (initial orthostatic hypotension, classical orthostatic hypotension, delayed orthostatic hypotension) [25]. Such an approach enables a detailed assessment of adaptive responses and differences in cardiovascular regulation between older adults and younger adults (Figure 2).

### 2.10. Statistical Analysis

The normality of the data was assessed using the Shapiro–Wilk test. Continuous variables (e.g., SBP, DBP) with a parametric distribution were reported as mean ± SD, along with the 95% confidence interval (95% CI). Non-parametric continuous variables were reported as median with interquartile range (IQR) and minimum and maximum values. For sample characterization, continuous data (e.g., height, body mass) were presented as mean ± SD, 95% CI, minimum, and maximum values, while categorical data (e.g., sex, educational level) were reported as absolute numbers and relative percentages. The Chi-square test was used to compare categorical variables.

For comparison data between groups (younger vs. older), the Mann–Whitney U test was applied. To adjust for multiple hypothesis testing, the *p*-values were corrected using the Benjamini–Hochberg adjustment [28], with the false discovery rate (FDR) set at 0.05. Significant results were reported based on the corrected *p*-values.

For continuous variables, effect sizes were calculated to quantify the magnitude of between-group differences. For parametric data, Cohen’s d was used [29]; for non-parametric data [30] the correlation coefficient r was applied. Effect size was classified as low (≤0.05), medium (0.06–0.25), high (0.26–0.50), and very high (>0.50) [29]. Correlations between CO and SVR at 0–30 s (phase 1), 30–60 s (phase 2), 60–180 s (phase 3), and 300–420 s (phase 4) were analyzed using Pearson or Spearman correlation, depending on the data distribution. Correlation magnitudes were interpreted as small (r = 0.10–0.29), medium (r = 0.30–0.49), and large (r ≥ 0.50) [29]. All analyses were conducted using R^®^ (Version 4.1.1, Vienna, Austria), with statistical significance set at α < 0.05.

## 3. Results

**Figure 3:**
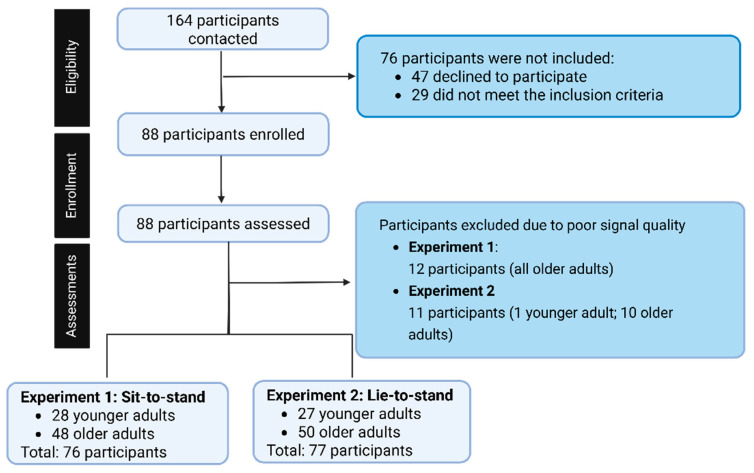
Recruitment flow chart of the study.

In the sit-to-stand transition, data from 12 participants were excluded from the analysis, where the final sample size was 28 younger adults and 48 older adults. In the lie-to-stand, 11 participants were excluded from the analysis, resulting in a final sample size of 27 younger adults and 50 older adults (Figure 3). Data was excluded due to poor signal quality (e.g., noisy, motion artifacts).

Among older adults, 70% were female and 30% were male, while the younger adult group had an even distribution (50% females and males). Anthropometric data showed that older adults had a higher BMI and were shorter than younger adults, but there were no statistically significant differences in body mass (Table 1). Regarding older adults’ cardiovascular medication, 27% of participants were on calcium channel blockers (*n* = 14), 13% on beta-blockers (*n* = 7), 10% on diuretics (*n* = 5), 8% on ACE inhibitors (*n* = 4), and 8% on angiotensin receptor blockers (*n* = 4). Also, only two of the younger adults (7%) were on psychotropic medications.

### 3.1. Sit to Stand Responses

During sit-to-stand, at baseline, older adults exhibited higher SBP, DBP, MAP, and SVR, while HR, CO, and SV were lower compared to younger adults. Immediately on sit-to-stand, older adults experienced a greater drop in SBP, DBP, MAP, SVR, lower HR and CO peaks, and larger SV peak values compared to younger adults. Additionally, SBP, DBP, and MAP short-term cardiovascular compensatory responses showed no statistically significant differences between older adults and younger adults during phases 1 (0–30 s) and 2 (30–60 s). However, older adults exhibited higher values in phases 3 (60–180 s) and 4 (300–420 s), except for DBP. Older adults also had lower SVR values in phases 1 and 2, but no differences in phases 3 and 4. In older adults, HR had lower values and SV had higher values across all phases compared to younger adults. CO values were lower in phases 1 and 4, but no differences were observed in phases 2 and 3 (Table 2).

**Figure 4:**
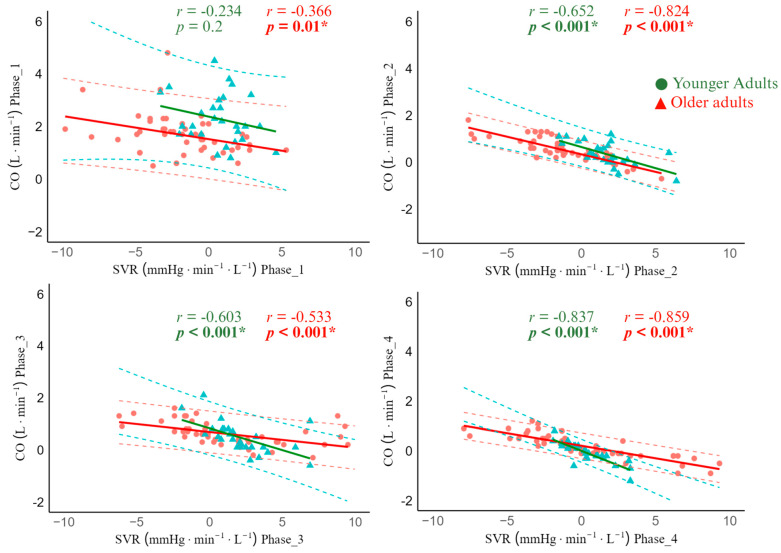
Cardiac output and systemic vascular resistance correlation during sit-to-stand in younger adults and older adults. Phase 1: 0–30 s; Phase 2: 30–60 s; Phase 3: 60–180 s; Phase 4: 300–420 s. Red and blue dashed lines represent the 95% confidence interval. * Statistically significant correlation between CO and SVR.

Regarding ΔCO-ΔSVR matching, in sit-to-stand, younger adults demonstrated a non-significant correlation in phase 1 (r = −0.234, 95% CI −0.569; 0.137, *p* = 0.2). However, a significant strong negative correlation was observed from phases 2–4 (phase 2: r = −0.652, 95% CI −0.825; −0.369, *p* < 0.001, phase 3: r = −0.603, 95% CI −0.797; −0.296, *p* < 0.001), and phase 4: r = −0.837, 95% CI −0.922; −0.674, *p* < 0.001). Older adults showed moderate and strong significant negative correlations between ΔCO and ΔSVR in all phases (phase 1: r = −0.366, 95% CI −0.534; −0.011, *p* = 0.01, phase 2: r = −0.824, 95% CI −0.898; −0.704, *p* < 0.001), phase 3: r = −0.533, 95% CI −0.710; −0.293, *p* < 0.001), and phase 4: r = −0.859, 95% CI −0.919; −0.761, *p* < 0.001) (Figure 4).

### 3.2. Lie to Stand Responses

During lie-to-stand, older adults exhibited higher SBP, DBP, MAP, and SVR, while CO and SV were lower compared to younger adults, with no differences in HR. Older adults experienced a greater drop in SBP, DBP, MAP, and SVR, lower HR peak, higher SV peak, and no differences in CO peak values (Table 3). Furthermore, older adults’ SBP, DBP, MAP, and SVR short-term compensatory responses were lower in phases 1 and 2, but similar in phases 3 and 4 than younger adults. However, HR values were lower, and SV values were higher in phases 1–3 but not in phase 4 in older adults. No statistically significant differences were observed in CO across all phases between older adults and younger adults (Table 3).

**Figure 5:**
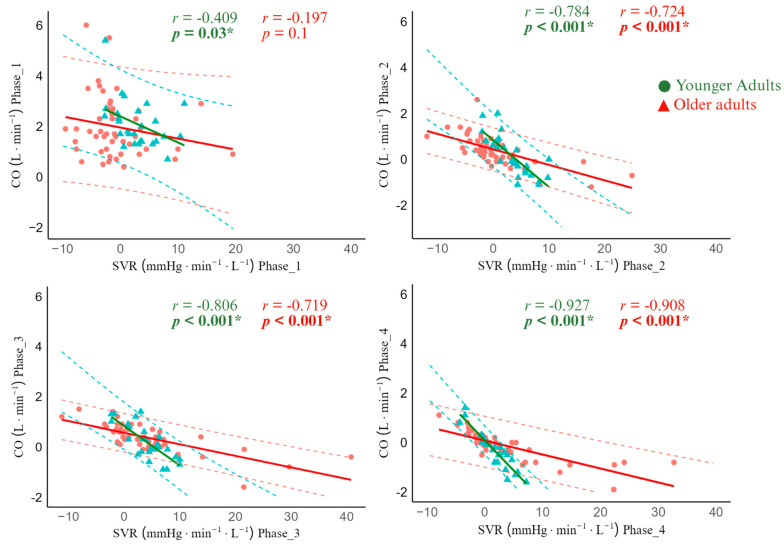
Cardiac output and systemic vascular resistance correlation during lie-to-stand transition in younger adults and older adults. Phase 1: 0–30 s; Phase 2: 30–60 s; Phase 3: 60–180 s; Phase 4: 300–420 s. Red and blue dashed lines represent the 95% confidence interval. * Statistically significant correlation between CO and SVR.

In lie-to-stand, there was moderate and strong significant negative correlations between ΔCO and ΔSVR across all phases in younger adults (phase 1: r = −0.409, 95% CI −0.683; −0.034, *p* = 0.03), phase 2: r = −0.784, 95% CI −0.897; −0.575, *p* < 0.001), phase 3: r = −0.806, 95% CI −0.908; −0.614, *p* < 0.001), and phase 4: r = −0.927,95% CI −0.966; −0.844, *p* < 0.001). Unlike younger adults, the ΔCO and ΔSVR correlation was not statistically significantly different in older adults in phase 1 (r = −0.197, 95% CI −0.430; 0.118, *p* = 0.1), but strong statistically significant in phase 2 (r = −0.724, 95% CI −0.804; −0.486, *p* < 0.001), phase 3 (r = −0.719, 95% CI −0.834; −0.553, *p* < 0.001), and phase 4 (r = −0.908, 95% CI −0.824; −0.531, *p* < 0.001) (Figure 5).

## 4. Discussion

Although previous studies have examined the effect of age on short-term cardiovascular compensatory responses [11,12,31], our study is the first to analyze phase-specific cardiovascular responses, including the initial, early, late, and delayed periods, in younger and older adults during both sit-to-stand and lie-to-stand transitions. The main findings indicate that immediately on standing, older adults experienced a larger drop in SBP, DBP, MAP, and SVR, as well as blunted increases in HR and CO, but exhibited higher SV responses. Older adults had attenuated short-term compensatory responses in phases 1 (initial) and 2 (early) across SBP, DBP, MAP, SVR, CO, and HR. They also showed a lower peak HR and CO response, alongside a larger SV compensatory response throughout phases 1 to 4. Regarding the matching between ΔCO and ΔSVR, which reflects the timing of BP regulation, older adults exhibited significant negative correlations across all phases (1–4) during sit-to-stand and phases 2–4 during lie-to-stand transition. These findings indicated that under lower gravitational stress, older adults were able to regulate BP within the expected 30 s of standing, but younger adults could not. However, during the lie-to-stand transition (higher gravitational stress), significant negative correlations were observed in phases 2–4, indicating that older adults require more time (30–60 s) to regulate BP effectively.

### 4.1. Immediate Cardiovascular Responses to Active Standing

Immediately on standing, older adults exhibited greater drops in SBP, DBP, and MAP in both transitions compared to younger adults. These results are partially consistent with previous studies [15], but in contrast with Kawaguchi et al. [13]. Smith and Fasler [15] observed that older adults had a larger SBP drop compared to younger adults, with no differences in DBP. Kawaguchi et al. [13] reported no statistically significant differences in SBP, DBP, and MAP drop during sit-to-stand between the groups.

In the current study, SVR drop (the lowest point reached on standing) was more pronounced in older adults (sit-to-stand: 44%; lie-to-stand: 45%) than in younger adults (sit-to-stand: 33%; lie-to-stand: 33%) following the active standing orthostatic stress. This finding contrasts with the results of Vargas et al. [11], who reported that older adults had a smaller percentage change in SVR from baseline to 70° head-up tilt (22 ± 27%) compared to younger adults (62 ± 33%). It also differs from Wieling et al. [12], who reported no statistical differences in SVR changes during lie-to-stand (active stand) between older adults (17 ± 24%) and younger adults (25 ± 10%).

Older adults also exhibited a blunted peak HR response compared to younger adults in both conditions, consistent with previous findings [11,12,14]. Wieling et al. [12] reported age-group differences in HR one minute after lying to standing. Vargas et al. [11] observed smaller percentage HR changes in older adults after a 70° head-up tilt. Smith et al. [14] showed attenuated HR increases in older males across multiple tilt angles. This study also reported that older adults had a reduced peak CO during sit-to-stand, but not lie-to-stand, aligning with studies reporting no significant age-related differences in peak CO [12]. In contrast, older adults demonstrated higher SV peaks during both transitions, consistent with earlier findings [11,12].

These cardiovascular differences likely reflect age-related changes, including arterial stiffness, impaired vascular compliance, slower baroreflex responses, reduced β-adrenergic sensitivity, slower parasympathetic withdrawal, and impaired baroreflex function [7,32,33]. Although baseline SV was lower, older adults reached higher SV peaks, likely a compensatory mechanism to offset reduced BP, CO, and delayed vasoconstriction [7,33]. Overall, the data indicate that older adults have an impaired ability to regulate BP immediately on standing, particularly under higher orthostatic stress challenges.

### 4.2. Short-Term Cardiovascular Compensatory Responses Across Phases During Active Standing Orthostatic Stress

The short-term cardiovascular compensatory phases (1–4) and expected physiological responses are described in Figure 2. To date, no study has directly compared the short-term time course of cardiovascular compensation between older and younger adults in all these phases following lower (sit-to-stand) and higher (lei-to-stand) gravitational orthostatic stress. Therefore, this discussion primarily draws on a limited number of studies that examined these responses separately by age group, as well as studies involving passive maneuvers (head-up tilt), despite the inherent differences between active and passive postural transitions.

In this study, SBP, DBP, and MAP responses during sit-to-stand did not differ between older and younger adults in the first 30 s (phase 1). However, during lie-to-stand, older adults showed slower BP recovery, requiring more than 30 s (phase 2) to return to initial values. Consistent with our results, Ten Harkel et al. [34] reported that SBP and DBP returned to baseline within 20 s in younger males (22–40 years old) after lie-to-stand. Finucane et al. [10] investigated 4475 middle-aged and older adults (62.8 ± 9.2 years) during lie-to-stand and reported delayed SBP stabilization, within 20–30 s in males and 30 s in females aged 50–69 years, but up to 60 s in males and 90 s in females aged ≥ 70 years. Similarly, DBP stabilization occurred within 40 s for those aged 50–69 years but did not return to baseline in those aged ≥ 70 years.

In the current study, during phases 1 (0–30 s) and 2 (30–60 s), older adults exhibited diminished SVR response compared to younger adults in both conditions, consistent with previous studies [14,32]. Additionally, older adults demonstrated a blunted HR short-term compensatory response across all phases during both active standing orthostatic stress, consistent with the findings from Wieling et al. [12] and Kim et al. [31]. Wieling et al. [12] reported lower HR in older adults at 1, 2, and 5 min after lie-to-stand, while Kim et al. [31] observed smaller HR increases in older adults at 5 min after standing. In contrast, CO responses in the present study were similar between groups across all phases. Interestingly, older adults showed a higher SV compensatory response in all phases (1 to 4) of sit-to-stand and in phases 1 to 3 of lie-to-stand. Partially agreeing with our results, Wieling et al. [12] found no age-related differences in CO percentage changes at 1 and 2 min after lie-to-stand, although they did not report a greater CO drop in older adults at 5 min. The authors also reported a smaller SV percentage drop in older adults at 1, 2, and 5 min of standing.

### 4.3. Cardiac Output and Systemic Vascular Resistance Matching Responses During Active Standing Orthostatic Stress

During postural transitions, the coordination/matching between ΔCO and ΔSVR is essential for maintaining MAP and ensuring adequate cerebral blood flow [7,33]. This is the first study to examine the correlation between ΔCO and ΔSVR across specific phases, aiming to determine the timing of BP regulation relative to baseline values. Given the novelty of this analysis, the discussion focuses on the physiological aspects of cardiovascular responses. During sit-to-stand, older adults exhibited a significant negative correlation between CO and SVR across all phases, whereas younger adults showed this correlation from phases 2 to 4. In lie-to-stand, older adults showed a significant negative correlation from phases 2 to 4, while younger adults displayed this correlation across all phases.

These findings indicate that older adults regulate BP within 30 s (phase 1) during sit-to-stand, demonstrating that they can still compensate under a lower gravitational stress challenge, unlike younger adults, who took longer to regulate BP in lower gravitational stress. This delayed regulation in sit-to-stand in younger adults may be partially explained by their greater vascular compliance, which facilitates peripheral blood pooling and may delay venous return and subsequent BP recovery in lower gravitational stress [33]. Additionally, the relatively lower gravitational stress of the sit-to-stand transition may not be sufficient to provoke substantial autonomic or hemodynamic modulation in individuals with preserved cardiovascular regulatory capacity [35]. Another factor that may partially explain this response in younger adults is the speed of transition. Younger adults likely stand up more quickly than older adults, which may require slightly more time for the cardiovascular system to adjust due to the rapid gravitational shift [36,37], a factor that may not affect older adults in the same way. O’Connor et al. [37] demonstrated that standing speed significantly influences both peripheral and central hemodynamic responses, demonstrating the importance of standardized test protocols. Their findings may partially explain some of our results, as older adults generally perform sit-to-stand and lie-to-stand transitions more slowly than younger adults. While we acknowledge that standardization of postural transitions is important for methodological consistency, it may also reduce external and ecological validity, since natural variations in standing speed are characteristic of daily life.

In contrast, in lie-to-stand, older adults exhibit delayed BP regulation, achieving matching between ΔCO-ΔSVR in phase 2 (30–60 s). This delayed response may reflect the diminished efficiency of cardiovascular and autonomic compensatory responses, including slower baroreflex activation [7], delayed vasoconstriction [33], and reduced autonomic response [38]. Consequently, older adults exhibited impaired rapid responses to counteract the sudden BP drop and increased blood pooling in the lower extremities under higher orthostatic stress.

The current findings highlight that impaired compensatory responses in older adults are particularly evident during distinct short-term intervals and dependent on gravitational orthostatic stress (lower vs. higher). This temporal perspective has important clinical implications. Conventional orthostatic testing may underestimate risk by overlooking these brief but critical windows of vulnerability (hypotension), during which cerebral hypoperfusion and fall risk may be highest. Our results suggest that targeted preventive strategies could be optimized by timing their application to these vulnerable intervals. For example, teaching older adults to perform physical counter-maneuvers immediately upon standing (e.g., leg crossing with muscle tensing or tip-toe standing) to improve venous return may help counteract early blood pressure drops and reduce the likelihood of dizziness or falls. Thus, interval-based analysis not only advances mechanistic understanding but also provides a rationale for refining diagnostic approaches and tailoring interventions in this population.

### 4.4. Limitations

This study has limitations that should be considered when interpreting the findings. The small sample size limited the study’s generalizability. Medication used was recorded but not controlled, which could have influenced cardiovascular responses during active orthostatic stress. Many older adults take antihypertensives, beta-blockers, or diuretics, which may affect HR, BP, and vascular resistance [39]. While these medications may have impacted compensatory responses under investigation, they also reflect real-world conditions, making the findings more applicable to the community-dwelling older population.

In young female participants, the menstrual cycle phase was not controlled. However, its impact on cardiovascular responses remains controversial in the literature. Studies have suggested that estrogen levels influence vascular tone [7,40] and hemodynamic responses during postural transitions [41,42]. Conversely, other studies report no significant effects on hemodynamic responses [43,44]. Therefore, whether and how the menstrual cycle affected our results, and the extent of this influence, remains uncertain.

Sex distribution is another limitation that must be considered when interpreting our findings. Sachse et al. [44] demonstrated sex-related differences in cardiovascular responses to orthostatic challenge among older adults, and the unequal distribution of sexes in our study groups may have influenced our results. Future research should aim for more balanced samples to control age influences in cardiovascular responses. Sex and frailty are potential confounders that should be acknowledged as limitations. Future research should explore how these factors impact cardiovascular responses during active standing orthostatic stress. 

## 5. Conclusions

Older adults experienced a more pronounced drop in BP and SVR, along with delayed short-term compensatory responses compared to younger adults, indicating impairments in cardiovascular regulation. They also showed a blunted HR compensatory response during both transitions. Despite this, SV was higher in older adults, indicating a greater reliance on SV as a compensatory response to attenuating BP drops. Regarding BP stabilization, older adults required more time under higher gravitational orthostatic stress, achieving regulation in phase 2 (30–60 s) after standing, but not under lower gravitational stress, which occurred in phase 1 (0–30 s). Overall, our findings highlight that older adults exhibit delayed and less effective short-term cardiovascular responses during active standing, which may contribute to increased risk of orthostatic hypotension, orthostatic intolerance, or falls in daily life.

## Figures and Tables

**Table 1:** Sample characteristics of younger adults and older adults.

Anthropometry							
	**Younger Adults**			**Older Adults**			
**Variable**	**Mean ± SD**	**CI 95%**	**Min; Max**	**Mean ± SD**	**CI 95%**	**Min; Max**	***p*** **-Value**
Age (years)	21.0 ± 2.3	20.8; 22.6	18.0; 28.0	70.5 ± 3.9 ↑	69.3; 71.5	63.0; 78.0	<0.001 *
Height (m)	1.73 ± 0.07	1.69; 1.76	1.56; 1.86	1.64 ± 0.08 ↓	1.62; 1.66	1.46; 1.84	<0.001 *
Body mass	67.6 ± 11.9	62.9–72.2	42.6; 90.5	73.7 ± 15.4	69.4; 78.0	42.5; 110.0	0.07
BMI (kg/m^2^)	22.6 ± 3.38	21.3–23.9	16.5; 30.4	27.3 ± 5.6 ↑	25.8; 28.9	17.1; 48.4	<0.001 *
	**Younger Adults**			**Older Adults**			***p*** **-Value**
**Hypertension**	*n*	%	----	*n*	%	----	----
	0	0		20	40		<0.001 *
**Diabetes**	*n*	%	----	*n*	%	----	
	0	0		2	4		0.53
**Medications**	*n*	%	----	*n*	%	----	
Cardiovascular	0	0	----	20	40	----	<0.001 *
Psychotropic	2	7	---	7	14	----	0.47

Mean ± Standard deviation (SD); *n*: number of participants; 95% CI: 95% confidence interval; Min-Max: Minimum; Maximum; BMI: body mass index; ↑ higher than; ↓ lower than. * Statistically significant differences between younger and older adults.

**Table 2:** Comparison of baseline, amplitude, and short-term cardiovascular compensatory responses during sit-to-stand between younger adults and older adults across phases.

Variable	Group	Baseline	Amplitude	Phase 1	Phase 2	Phase 3	Phase 4
SBP (mmHg)	Younger adults	111.6 ± 9.1 (108; 115)	−8.4|6.4 (2.6; 43.8)	4.8|4.0 (−5.1; 22.0)	2.6 ± 4.6 (0.8; 4.4)	1.8|3.3 (−3.3; 20.0)	−1.0 ± 3.6 (−2.4; 0.4)
	Older adults	129.5 ± 12.9 ↑ (126; 133)	−20.4|11.0 ↑ (5.4; 41.0)	2.7|9.7 (−15.7; 21.9)	3.3 ± 1.1 (5.5; 7.6)	2.1|7.8 ↑ (−15.0; 22.4)	3.4 ± 2.2 ↑ (1.5; 5.3)
	**Adj *p***	0.003 *	0.003 *	0.23	0.6	0.003 *	0.003 *
	ES	1.5	1.1	−0.3	0.1	0.76	0.8
DBP (mmHg)	Younger adults	76|6 (54; 82)	−7.8|4.4 (3.4; 28.3)	2.8|3.0 (−9.7; 8.2)	1.5|2.7 (−8.5; 6.7)	2.3|2.7 (−8.9; 11.4)	1.1|2.2 (−12.0; 6.2)
	Older adults	80|10 ↑ (56; 92)	−13.0|6.7 ↑ (5.5; 27.7)	0.6|3.8 (−11.8; 7.6)	1.2|3.0 (−9.9; 10.9)	3.3|37 (−9.3; 13.6)	2.0|4.1 (−11.6; 11.3)
	**Adj *p***	0.029 *	0.035 *	0.23	0.30	0.11	0.2
	ES	0.3	0.5	0.3	0.1	0.11	0.2
MAP (mmHg)	Younger adults	87 ± 5.05 (85.1; 89)	−7.7|4.4 (3.4; 28.3)	2.1|3.6 (−6.3; 10.4)	2.8|3.0 (−9.7; 8.2)	2.4|2.7 (−8.2; 11.7)	−0.1|4.3 (−13.7; 3.9)
	Older adults	95.5 ± 8.52 ↑ (93.2; 97.9)	−13.3|6.7 ↑ (5.5; 27.7)	1.3|4.3 (−12.0; 9.1)	0.6|3.8 (−11.8; 7.8)	3.3|3.7 ↑ (−9.3; 13.6)	1.9|4.3 ↑ (−12.6; 10.6)
	**Adj *p***	0.002 *	0.023 *	0.11	0.9	0.012 *	0.008 *
	ES	1.1	0.7	0.2	0.01	0.36	0.4
SVR (mmHg·min^−1^·L^−1^)	Younger adults	20.6|6.5 (13.8; 31.1)	−7.0|2.7 (2.7; 11.6)	0.7 ± 1.8 ↑ (−0.0; 1.4)	1.6 ± 1.7 (0.9; 2.3)	1.7|2.1 (−1.8; 6.9)	0.2|1.7 (−1.9; 3.3)
	Older adults	25.8|8.4 ↑ (18.2; 49.8)	−11.3|6.6 ↑ (5.3; 21.5)	−2.0 ± 3.3 (−2.9; −1.0)	−1.3 ± −2.1 ↓ (−0.5; 2.7)	0.5|5.7 (−6.1; 11.4)	−0.4|5.4 (−7.9; 9.3)
	**Adj *p***	0.0017 *	0.0017 *	0.0017 *	0.0017 *	0.23	0.4
	ES	0.6	0.7	−0.9	−1.2	0.1	0.1
HR (bpm)	Younger adults	71.9 ± 10.2 (68.0; 75.9)	29.2 ± 5.3 (27.1; 31.3)	29.2 ± 5.3 (27.1; 31.3)	11.7 ± 8.3 (8.5; 15.0)	18.0 ± 7.8 (15.0; 21.1)	14.1 ± 5.5 (11.9; 16.3)
	Older adults	64.5 ± 61.8 ↓ (67.1; 9.0)	12.3 ± 4.4 ↓ (11.0; 13.6)	12.3 ± 4.4 ↓ (11.0; 13.6)	7.0 ± 5.9 ↓ (8.1; 3.8)	9.3 ± 3.9 ↓ (8.1; 10.5)	7.5 ± 4.2 ↓ (6.2; 8.7)
	**Adj *p***	0.001 *	0.001 *	0.001 *	0.001 *	0.001 *	0.001 *
	ES	−0.8	−3.5	−3.5	−0.8	−1.5	−1.4
CO (L·min^−1^)	Younger adults	5.4 ± 1.2 (5.0; 6.0)	2.0|1.3 (0.7; 4.5)	2.0|1.3 (0.7; 4.5)	0.3 ± 0.4 (0.1; 0.6)	0.4 ± 0.5 (0.2; 0.7)	−0.1 ± 0.4 (−0.2; 0.1)
	Older adults	3.5 ± 0.8 ↓ (3.3; 3.8)	1.6|0.8 ↓ (0.5; 4.7)	1.6|0.8 ↓ (0.5; 4.7)	2.2 ± 0.9 (1.9; 2.6)	0.5 ± 0.4 (0.4; 0.7)	0.1 ± 0.4 ↑ (0.0; 0.3)
	**Adj *p***	0.035 *	0.005 *	0.005 *	0.11	0.4	0.028 *
	ES	−1.9	0.4	0.4	0.4	0.2	0.5
SV (mL)	Younger adults	77.3 ± 18.3 (70.2; 84.4)	9.1 ± 7.8 (6.0; 12.2)	−3.7|9.2 (−30.8; 14.0)	−8.1 ± 7.1 (−10.9; −5.3)	−10.0|1.1 (8.6; −28.7)	−22.3|11.5 (−46.9; −7.5)
	Older adults	55.9 ± 14.4 ↓ (51.7; 60.1)	13.6 ± 8.1 ↑ (11.2; 16.0)	3.8|9.5 ↑ (−7.2; 53.1)	0.1 ± 8.1 ↑ (−2.2; 2.5)	−3.9|7.4 ↑ (−25.8; 28.3)	−15.0|12.2 ↑ (−42.6; 30.0)
	**Adj *p***	0.0014 *	0.02 *	0.0014 *	0.0014 *	0.0014 *	0.011 *
	ES	−1.3	0.6	0.5	1.0	0.6	0.35

Mean ± Standard Deviation (SD) with (95% Confidence Interval for the mean); |: Interquartile range (IQR) with (Minimum and Maximum); SBP: systolic blood pressure; DBP: diastolic blood pressure; MAP: mean arterial pressure; SVR: systemic vascular resistance; HR: heart rate; CO: cardiac output; SV: stroke volume; Adj *p*: adjusted *p*-value; ES: effect size. ↓ lower than; ↑ higher than. Phase 1: 0–30 s; Phase 2: 30–60 s; Phase 3: 60–180 s; Phase 4: 300–420 s. * Statistically significant differences between younger adults and older adults. The variable values are expressed as absolute numbers in baseline and Δ from amplitude to phase 4, where negative numbers represent below baseline values and positive numbers above baseline values.

**Table 3:** Comparison of baseline, amplitude, and short-term cardiovascular compensatory responses during lie-to-stand between younger and older adults across phases.

Variable	Group	Baseline	Amplitude	Phase 1	Phase 2	Phase 3	Phase 4
SBP (mmHg)	Younger adults	111.6 ± 9.1 (108; 115)	−15.1 ± 6.6 (12.4; 17.7)	5.9 ± 4.9 (4.0; 7.9)	3.2|6.0 (−6.4; 9.7)	2.5|4.1 (−2.2; 8.3)	−0.9|3.5 (−4.1; 4.2)
	Older adults	129.5 ± 12.9 ↑ (126; 133)	−31.7 ± 12.4 ↑ (28.1; 35.3)	−2.5 ± 10.7 ↓ (−15.7; 21.9)	−1.2|9.0 ↓ (−29.1; 21.4)	2.6|8.0 (−23.2; 27.1)	−1.2|11.9 (−32.8; 19.9)
	**Adj *p***	0.003 *	0.0035 *	0.035 *	0.007 *	0.5	0.23
	ES	1.5	1.5	−0.9	0.4	0.1	0.2
DBP (mmHg)	Younger adults	76|6 (54; 82)	−11.2|4.0 (2.5; 17.9)	4.0|3.8 (−2.6; 14.0)	3.0|3.1 (−2.4; 10.6)	4.3|2.8 (0.6; 14.0)	2.4|2.0 (−0.3; 10.3)
	Older adults	80|10 ↑ (56; 92)	−19.5|5.9 ↑ (0.0; 34.6)	0.1|7.0 ↓ (−9.8; 21.7)	1.2|5.8 ↓ (−10.0; 27.1)	2.5|6.6 (−7.6; 32.5)	2.8|6.0 (−8.2; 28.3)
	**Adj *p***	0.029 *	0.0017 *	0.0017 *	0.004 *	0.46	0.9
	ES	0.3	0.8	0.6	0.4	0.1	0.01
MAP (mmHg)	Younger adults	87 ± 5.05 (85.1; 89)	−10.7 ± 5.5 (9.2; 12.1)	4.2 ± 3.2 (3.0; 5.5)	3.0|3.1 (−2.4; 10.6)	4.4|2.8 (0.6; 4.0)	1.7|8.6 (−14.5; 21.9)
	Older adults	95.5 ± 8.52 ↑ (93.2; 97.9)	−19.8 ± 6.9 ↑ (17.8; 21.8)	0.5 ± 3.9 ↓ (−0.6; 1.6)	−1.6|7.3 ↓ (−16.8; 20.9)	2.6|6.6 (−7.6; 32.5)	2.2|8.6 (−14.5; 21.9)
	**Adj *p***	0.002 *	0.0014 *	0.0014 *	0.0014 *	0.58	0.90
	ES	1.1	1.8	−1.1	0.5	0.1	0.01
SVR (mmHg·min^−1^·L^−1^)	Younger adults	19.4 ± 3.6 (18.0; 20.9)	−6.4|3.2 (3.1; 2.3)	2.4|4.9 (−2.7; 11.0)	3.6|4.8 (−2.0; 10.0)	4.4|3.9 (−2.3; 10.0)	1.7|3.7 (−4.3; 7.2)
	Older adults	27.8 ± 7.0 ↑ (25.8; 29.8)	−12.5|5.2 ↑ (4.9; 25.3)	−1.7|4.1 ↓ (−13.1; 19.5)	−0.8|4.1 ↓ (−11.9; 24.9)	2.7|6.4 (−11.1; 40.8)	0.2|6.8 (−13.3; 40.5)
	**Adj *p***	0.0017 *	0.0017 *	0.0017 *	0.0017 *	0.14	0.7
	ES	1.4	0.9	0.6	0.5	0.2	0.03
HR (bpm)	Younger adults	66.4 ± 9.8 (62.5; 70.2)	35.4|8.1 ↑ (25.8; 53.7)	35.4|8.1 (25.8; 53.7)	16.7|12.5 (−1.9; 47.8)	21.7|12.0 (11.5; 44.7)	18.8|9.0 (3.8; 35.5)
	Older adults	62.6 ± 8.8 (60.0; 65.1)	16.2|5.8 (5.6; 40.4)	16.2|5.8 ↓ (5.6; 40.4)	10.9|6.8 ↓ (0.4; 41.3)	13.5|8.2 ↓ (4.5; 42.8)	9.1|7.1 ↓ (1.8; 32.1)
	**Adj *p***	0.08	0.0014 *	0.0014 *	0.0081 *	0.0014 *	0.0014 *
	ES	−0.4	0.9	0.9	0.4	0.7	0.7
CO (L·min^−1^)	Younger adults	5.5|1.3 ↑ (3.9; 9.0)	1.7|1.0 (0.7; 5.4)	1.7|1.0 (0.7; 5.3)	0.1 ± 0.8 (−0.2; 0.4)	0.1 ± 0.6 (−0.0; 0.4)	−0.2|0.8 (−1.5; 1.3)
	Older adults	3.4|1.0 (2.2; 5.6)	1.7|1.5 (0.3; 6.0)	1.7|1.5 (0.3; 6.0)	0.4 ± 0.6 (0.2; 0.6)	0.5 ± 0.5 (0.4; 0.7)	−0.0|0.7 (−2.7; 1.0)
	**Adj *p***	0.035 *	0.4	0.4	0.17	0.17	0.28
	ES	0.8	0.1	0.1	0.4	0.3	0.1
SV (mL)	Younger adults	85.3|22.9 ↑ (61.8; 122.2)	−0.1|16.7 (−27.7; 21.6)	−16.8|11.2 (−37.9; 3.1)	−19.6 ± 8.5 (−23.0; −16.3)	−23.1 ± 8.5 (−26.5; −19.7)	−19.4|16.4 (−42.6; −0.3)
	Older adults	55.7|14.9 (35.4; 93.1)	10.5|10.7 ↑ (−5.4; 49.7)	3.9|11.0 ↑ (−18.0; 33.8)	−5.5 ± 7.9 ↑ (−7.8; −3.2)	−8.4 ± 7.1 ↑ (−10.8; −6.0)	−17.5|24.4 (−55.7; 3.7)
	**Adj *p***	0.0011 *	0.0011 *	0.0011 *	0.0011 *	0.0011 *	0.8
	ES	0.8	0.5	0.85	1.7	1.7	0.03

Mean ± Standard Deviation (SD) with (95% Confidence Interval for the mean); |: Interquartile range (IQR) with (Minimum and Maximum); SBP: systolic blood pressure; DBP: diastolic blood pressure; MAP: mean arterial pressure; SVR: systemic vascular resistance; HR: heart rate; CO: cardiac output; SV: stroke volume; Adj *p*: adjusted *p*-value; ES: effect size. ↓ lower than; ↑ higher than. Phase 1: 0–30 s; Phase 2: 30–60 s; Phase 3: 60–180 s; Phase 4: 300–420 s. * Statistically significant differences between younger adults and older adults. The variable values are expressed as absolute numbers in baseline and Δ from amplitude to phase 4, where negative numbers represent below baseline values and positive numbers above baseline values.

## Data Availability

The raw data supporting the conclusions of this article will be made available by the authors on request.

## Abbreviations

ACE	Angiotensin-Converting Enzyme
ARB	Angiotensin Receptor Blocker
BMI	Body Mass Index
BP	Blood Pressure
CI	Confidence Interval
CO	Cardiac Output
CO-SVR	Cardiac Output–Systemic Vascular Resistance Matching
CSEP	Canadian Society for Exercise Physiology
DBP	Diastolic Blood Pressure
ECG	Electrocardiogram
FDR	False Discovery Rate
HR	Heart Rate
IQR	Interquartile Range
MAP	Mean Arterial Pressure
OA	Older Adults
OH	Orthostatic Hypotension
OI	Orthostatic Intolerance
SBP	Systolic Blood Pressure
SD	Standard Deviation
STROBE	Strengthening the Reporting of Observational Studies in Epidemiology
SV	Stroke Volume
SVR	Systemic Vascular Resistance
YA	Younger Adults

## References

[1] de Matos D.G., de Santana J.L., Aidar F.J., Cornish S.M., Giesbrecht G.G., Mendelson A.A., Duhamel T.A., Villar R. (2025). Cardiovascular Regulation during Active Standing Orthostatic Stress in Older Adults Living with Frailty: A Systematic Review. Arch. Gerontol. Geriatr..

[2] de Matos D.G., de Santana J.L., Mendelson A.A., Duhamel T.A., Villar R. (2023). Integrated Dynamic Autonomic and Cardiovascular Regulation during Postural Transitions in Older Adults Living with Frailty: A Systematic Review Protocol. Int. J. Environ. Res. Public Health.

[3] van Wijnen V.K., Ten Hove D., Finucane C., Wieling W., van Roon A.M., Ter Maaten J.C., Harms M.P.M. (2018). Hemodynamic Mechanisms Underlying Initial Orthostatic Hypotension, Delayed Recovery and Orthostatic Hypotension. J. Am. Med. Dir. Assoc..

[4] Dani M., Dirksen A., Taraborrelli P., Panagopolous D., Torocastro M., Sutton R., Lim P.B. (2021). Orthostatic Hypotension in Older People: Considerations, Diagnosis and Management. Clin. Med. J. R. Coll. Physicians Lond..

[5] van Twist D.J.L., Mostard G.J.M., Sipers W.M.W.H. (2020). Delayed Recovery from Initial Orthostatic Hypotension: An Expression of Frailty in the Elderly. Clin. Auton. Res..

[6] Christopoulos E.M., Tran J., Hillebrand S.L., Lange P.W., Iseli R.K., Meskers C.G.M.M., Maier A.B. (2021). Initial Orthostatic Hypotension and Orthostatic Intolerance Symptom Prevalence in Older Adults: A Systematic Review. Int. J. Cardiol. Hypertens..

[7] Wehrwein E.A., Joyner M.J. (2013). Regulation of Blood Pressure by the Arterial Baroreflex and Autonomic Nervous System. Handb. Clin. Neurol..

[8] Ricci F., De Caterina R., Fedorowski A. (2015). Orthostatic Hypotension: Epidemiology, Prognosis, and Treatment. J. Am. Coll. Cardiol..

[9] Fedorowski A., Ricci F., Hamrefors V., Sandau K.E., Hwan Chung T., Muldowney J.A.S., Gopinathannair R., Olshansky B. (2022). Orthostatic Hypotension: Management of a Complex, But Common, Medical Problem. Circ. Arrhythmia Electrophysiol..

[10] Finucane C., O’Connell M.D.L., Fan C.W., Savva G.M., Soraghan C.J., Nolan H., Cronin H., Kenny R.A. (2014). Age-Related Normative Changes in Phasic Orthostatic Blood Pressure in a Large Population Study: Findings from the Irish Longitudinal Study on Ageing (TILDA). Circulation.

[11] Vargas E. (1983). Physiological Responses To Postural Change Young And Old Healthy Individuals In The Sydrome of Postural Hypotension Increases in Incidence with Increasing Age so That up to 20070 of Old People Suffer from the Syndrome. Exp. Gerontol..

[12] Wieling W., Veerman D.P., Dambrink J.H.A., Imholz B.P.M. (1992). Disparities in Circulatory Adjustment to Standing between Young and Elderly Subjects Explained by Pulse Contour Analysis. Clin. Sci..

[13] Kawaguchi T., Uyama O., Konishi M., Nishiyama T., Iida T. (2001). Orthostatic Hypotension in Elderly Persons during Passive Standing: A Comparison with Young Persons. J. Gerontol. Ser. A Biol. Sci. Med. Sci..

[14] Smith J.J., Hughes C.V., Ptacin M.J., Barney J.A., Tristani F.E., Ebert T.J. (1987). The Effect of Age on Hemodynamic Response to Graded Postural Stress in Normal Men. J. Gerontol..

[15] Smith S.A., Fasler J.J. (1983). Age-Related Changes in Autonomic Function: Relationship with Postural Hypotension. Age Ageing.

[16] Wieling W., van Twist D.J.L., van Wijnen V.K., Harms M.P.M. (2021). Spectrum of Hemodynamic Responses in the First 60 Seconds after Active Standing Up: Importance of Time Course of Blood Pressure Changes and Definitions. J. Am. Med. Dir. Assoc..

[17] Finucane C., O’Connell M.D.L., Donoghue O., Richardson K., Savva G.M., Kenny R.A. (2017). Impaired Orthostatic Blood Pressure Recovery Is Associated with Unexplained and Injurious Falls. J. Am. Geriatr. Soc..

[18] von Elm E., Altman D.G., Egger M., Pocock S.J., Gøtzsche P.C., Vandenbroucke J.P. (2014). The Strengthening the Reporting of Observational Studies in Epidemiology (STROBE) Statement: Guidelines for Reporting Observational Studies. Int. J. Surg..

[19] Canadian Society for Exercise Physiology, CSEP (2011). Canadian Physical Activity Guidelines for Older Adults 65 Years and Older.

[20] Guelen I., Westerhof B.E., Van Der Sar G.L., Van Montfrans G.A., Kiemeneij F., Wesseling K.H., Bos W.J.W. (2003). Finometer, Finger Pressure Measurements with the Possibility to Reconstruct Brachial Pressure. Blood Press. Monit..

[21] Finapres Medical Systems (2005). Finometer^TM^ User’s Guide.

[22] Faul F., Erdfelder E., Lang A.-G., Buchner A. (2007). G*Power 3: A Flexible Statistical Power Analysis Program for the Social, Behavioral, and Biomedical Sciences. Behav. Res. Methods.

[23] Van Der Velde N., Van Den Meiracker A.H., Stricker B.H.C., Van Der Cammen T.J.M. (2007). Measuring Orthostatic Hypotension with the Finometer Device: Is a Blood Pressure Drop of One Heartbeat Clinically Relevant?. Blood Press. Monit..

[24] Mol A., Slangen L.R.N., Trappenburg M.C., Reijnierse E.M., van Wezel R.J.A., Meskers C.G.M., Maier A.B. (2020). Blood Pressure Drop Rate after Standing up Is Associated with Frailty and Number of Falls in Geriatric Outpatients. J. Am. Heart Assoc..

[25] Romero-Ortuno R., Cogan L., Fan C.W., Kenny R.A. (2010). Intolerance to Initial Orthostasis Relates to Systolic BP Changes in Elders. Clin. Auton. Res..

[26] Gibbons C.H., Freeman R. (2006). Delayed Orthostatic Hypotension: A Frequent Cause of Orthostatic Intolerance. Neurology.

[27] Byun J.I., Moon J., Kim D.Y., Shin H., Sunwoo J.S., Lim J.A., Kim T.J., Lee W.J., Lee H.S., Jun J.S. (2018). Delayed Orthostatic Hypotension: Severity of Clinical Symptoms and Response to Medical Treatment. Auton. Neurosci. Basic. Clin..

[28] Yoav Benjamini and Yosef Hochberg (1995). Controlling the False Discovery Rate: A Practical and Powerful Approach to Multiple Testing. J. R. Stat. Soc. Ser. B (Methodol.).

[29] Cohen J. (1992). Statistical Power Analysis. Curr. Dir. Psychol. Sci..

[30] Kerby D.S. (2014). The Simple Difference Formula: An Approach to Teaching Nonparametric Correlation. Compr. Psychol..

[31] Kim Y.S., Bogert L.W.J., Immink R.V., Harms M.P.M., Colier W.N.J.M., Van Lieshout J.J. (2011). Effects of Aging on the Cerebrovascular Orthostatic Response. Neurobiol. Aging.

[32] Mohrman D.L.H. (2013). Cardiovascular Physiology.

[33] Ten Harkel A.D., Van Lieshout J.J., Van Lieshout E.J., Wieling W. (1990). Assessment of Cardiovascular Reflexes: Influence of Posture and Period Preceding Rest. Eurorehab.

[34] Saeidifard F., Medina-Inojosa J.R., Supervia M., Olson T.P., Somers V.K., Prokop L.J., Stokin G.B., Lopez-Jimenez F. (2020). The Effect of Replacing Sitting With Standing on Cardiovascular Risk Factors: A Systematic Review and Meta-Analysis. Mayo Clin. Proc. Innov. Qual. Outcomes.

[35] De Bruïne E.S., Reijnierse E.M., Trappenburg M.C., Pasma J.H., De Vries O.J., Meskers C.G.M., Maier A.B. (2017). Standing Up Slowly Antagonises Initial Blood Pressure Decrease in Older Adults with Orthostatic Hypotension. Gerontology.

[36] O’Connor J.D., O’Connell M.D.L., Nolan H., Newman L., Knight S.P., Kenny R.A. (2020). Impact of Standing Speed on the Peripheral and Central Hemodynamic Response to Orthostasis: Evidence from the Irish Longitudinal Study on Ageing. Hypertension.

[37] Wieling W., Karemaker J.M. (2013). Measurement of Heart Rate and Blood Pressure to Evaluate Disturbances in Neurocardiovascular Control. Auton. Fail..

[38] Freeman R., Abuzinadah A.R., Gibbons C., Jones P., Miglis M.G., Sinn D.I. (2018). Orthostatic Hypotension: JACC State-of-the-Art Review. J. Am. Coll. Cardiol..

[39] Stice J.P., Lee J.S., Pechenino A.S., Knowlton A.A. (2009). Estrogen, Aging and the Cardiovascular System. Future Cardiol..

[40] Fu Q., Vangundy T.B., Shibata S., Auchus R.J., Williams G.H., Levine B.D. (2010). Menstrual Cycle Affects Renal-Adrenal and Hemodynamic Responses during Prolonged Standing in the Postural Orthostatic Tachycardia Syndrome. Hypertension.

[41] Shankhwar V., Urvec J., Steuber B., Schmid Zalaudek K., Salon A., Hawliczek A., Bergauer A., Aljasmi K., Abdi A., Naser A. (2024). Effects of Menstrual Cycle on Hemodynamic and Autonomic Responses to Central Hypovolemia. Front. Cardiovasc. Med..

[42] Fu Q., Okazaki K., Shibata S., Shook R.P., Vangunday T.B., Galbreath M.M., Reelick M.F., Levine B.D. (2009). Menstrual Cycle Effects on Sympathetic Neural Responses to Upright Tilt. J. Physiol..

[43] Claydon V.E., Younis N.R., Hainsworth R. (2006). Phase of the Menstrual Cycle Does Not Affect Orthostatic Tolerance in Healthy Women. Clin. Auton. Res..

[44] Sachse C., Trozic I., Brix B., Roessler A., Goswami N. (2019). Sex Differences in Cardiovascular Responses to Orthostatic Challenge in Healthy Older Persons: A Pilot Study. Physiol. Int..

